# Whole genome sequencing distinguishes between relapse and reinfection in recurrent leprosy cases

**DOI:** 10.1371/journal.pntd.0005598

**Published:** 2017-06-15

**Authors:** Mariane M. A. Stefani, Charlotte Avanzi, Samira Bührer-Sékula, Andrej Benjak, Chloé Loiseau, Pushpendra Singh, Maria A. A. Pontes, Heitor S. Gonçalves, Emerith M. Hungria, Philippe Busso, Jérémie Piton, Maria I. S. Silveira, Rossilene Cruz, Antônio Schetinni, Maurício B. Costa, Marcos C. L. Virmond, Suzana M. Diorio, Ida M. F. Dias-Baptista, Patricia S. Rosa, Masanori Matsuoka, Maria L. F. Penna, Stewart T. Cole, Gerson O. Penna

**Affiliations:** 1 Tropical Pathology and Public Health Institute, Federal University of Goiás, Goiania, Goiás, Brazil; 2 Global Health Institute, École Polytechnique Fédérale de Lausanne, Switzerland; 3 University of Amazonas State, Manaus, Amazonas, Brazil; 4 Tropical Medicine Foundation Dr. Heitor Vieira Dourado, Manaus, Amazonas, Brazil; 5 Department of Medical Parasitology and Infection Biology, Swiss Tropical and Public Health Institute, Switzerland; 6 Department of Microbiology and Biotechnology Centre, Maharaja Sayajirao University of Baroda, Vadodara, India; 7 Dona Libânia Dermatology Centre, Fortaleza, Ceará, Brazil; 8 Tropical Dermatology and Venerology, Alfredo da Matta Foundation, Manaus, Amazonas, Brazil; 9 Faculty of Medicine, Federal University of Goiás, Goiania, Goiás, Brazil; 10 Lauro Souza Lima Institute, Bauru, São Paulo, Brazil; 11 Department of Mycobacteriology, Leprosy Research Center, National Institute of Infectious Diseases, Higashimurayama, Tokyo, Japan; 12 Jyu-kanbo National Museum, Kusatsu, Gunma, Japan; 13 Epidemiology and Biostatistics Department, Universidade Federal Fluminense, Rio de Janeiro, Brazil; 14 Tropical Medicine Centre, University of Brasília, Brasília DF, Brazil; 15 Fiocruz, Brasilia, Brazil; University of Tennessee, UNITED STATES

## Abstract

**Background:**

Since leprosy is both treated and controlled by multidrug therapy (MDT) it is important to monitor recurrent cases for drug resistance and to distinguish between relapse and reinfection as a means of assessing therapeutic efficacy. All three objectives can be reached with single nucleotide resolution using next generation sequencing and bioinformatics analysis of *Mycobacterium leprae* DNA present in human skin.

**Methodology:**

DNA was isolated by means of optimized extraction and enrichment methods from samples from three recurrent cases in leprosy patients participating in an open-label, randomized, controlled clinical trial of uniform MDT in Brazil (U-MDT/CT-BR). Genome-wide sequencing of *M*. *leprae* was performed and the resultant sequence assemblies analyzed *in silico*.

**Principal findings:**

In all three cases, no mutations responsible for resistance to rifampicin, dapsone and ofloxacin were found, thus eliminating drug resistance as a possible cause of disease recurrence. However, sequence differences were detected between the strains from the first and second disease episodes in all three patients. In one case, clear evidence was obtained for reinfection with an unrelated strain whereas in the other two cases, relapse appeared more probable.

**Conclusions/Significance:**

This is the first report of using *M*. *leprae* whole genome sequencing to reveal that treated and cured leprosy patients who remain in endemic areas can be reinfected by another strain. Next generation sequencing can be applied reliably to *M*. *leprae* DNA extracted from biopsies to discriminate between cases of relapse and reinfection, thereby providing a powerful tool for evaluating different outcomes of therapeutic regimens and for following disease transmission.

## Author summary

Leprosy, one of the most ancient human infectious diseases, affects skin and nerves and is caused by *Mycobacterium leprae* infection. Despite the effective use of multidrug therapy/MDT since the 80´s, over 200,000 new cases are reported yearly, indicating active transmission, especially in India and Brazil. Although rare, recurrent clinical manifestations after MDT can occur due to leprosy reactions, relapse by drug resistance, insufficient treatment or reinfection. Relapse and reinfection cannot be differentiated clinically and molecular genotyping of a predefined set of loci have limited resolution due to exceptional *M*. *leprae* genome conservation and low sequence diversity between strains from the same geographical area. This is the first report that has compared whole-genome sequences of *M*. *leprae* strains from original and recurrent leprosy episodes. *M*. *leprae* genome differences were detected between the strains from the first and second episodes in the three patients. In one patient, there was clear evidence for reinfection with an unrelated strain whereas the other two were considered true relapses due to minor strain differences. No known drug resistance mutations were detected, excluding drug resistance as the recurrence cause. Next generation sequencing of *M*. *leprae* DNA discriminates relapse from reinfection representing a powerful tool for evaluating different disease outcomes and transmission.

## Introduction

Leprosy is a complex dermato-neurologic and systemic disease[1] primarily caused by *Mycobacterium leprae* or to a much lesser extent by *Mycobacterium lepromatosis*.[2] Despite a strong decrease in leprosy prevalence since the systematic implementation of multidrug therapy (MDT) in the 1980’s, the incidence of disease, the major indicator of active transmission, remains high in many countries, especially in India and Brazil, showing that transmission continues unabated.[3] Overall, more than 200,000 new leprosy cases are reported each year worldwide.[3]

The MDT regimen for leprosy consists of different antibiotic combinations that are prescribed based on the number of skin lesions: a six-month regimen of rifampicin and dapsone for paucibacillary (PB) patients (<5 skin lesions) and a twelve month regimen of rifampicin, dapsone and clofazimine for multibacillary (MB) patients (>5 skin lesions).[4] In 2002, WHO proposed that a uniform MDT regimen (U-MDT) should be considered to treat all types of leprosy in order to facilitate leprosy control. In 2007, an open-label randomized and controlled clinical trial (uniform multidrug therapy for leprosy patients in Brazil, U-MDT/CT-BR) was initiated to compare U-MDT with the regular MDT for PB and MB patients.[5, 6] Clinical monitoring is still taking place with special emphasis on disease recurrence and leprosy type 1 and type 2 reactions (T1R/T2R).

An increased relapse rate and the possible emergence of drug resistance are major concerns for the shortened MDT proposal for MB patients. It is therefore important to address this issue by analyzing in depth all recurrent cases from the U-MDT/CT-BR trial. Molecular genotyping techniques, such as typing selected single nucleotide polymorphisms (SNP) or counting variable number tandem repeats (VNTR) have been used to differentiate reinfection from relapse.[7–11] However, the resolution of such techniques is often limited because of the exceptional level of genome conservation in *M*. *leprae* and the limited sequence diversity between strains from the same geographical area in particular.[12] In contrast, genome-wide approaches provide higher resolution and accuracy compared to genotyping based on a predefined set of loci, but are technically more complex. High throughput sequencing is becoming increasingly efficient and cost-effective with purified DNA but is more challenging with clinical specimens such as DNA extracted directly from skin biopsies, especially from formalin-fixed paraffin-embedded (FFPE) samples.

In this study, we investigated three recurrent cases of leprosy from the U-MDT/CT-BR trial to determine whether recurrence was due to drug resistance, bacterial persistence or to reinfection. To achieve this, we compared whole genome sequencing analysis of *M*. *leprae* collected from skin lesions at the initial diagnosis and during the recurrence of the disease and correlated the sequence data with the clinical, microbiologic and serologic findings.

## Methods

### Ethics statement

This study was approved by the regional research ethical committees, by the National Committee for Ethics in Research (CONEP, National Health Council/ Ministry of Health, Brazil, protocol # 001/06) and by the human and animal research ethics committee from the Federal University of Goiás (CEMHA/HC/UFG protocol # 166/2011). Written informed consent was obtained from all adult subjects and a parent or guardian of participants under the age of 18 years, provided informed consent on their behalf prior to inclusion in the study (ClinicalTrials.gov identifier: NCT00669643).

### Study design

Three recurrent cases of leprosy identified in the U-MDT/CT-BR trial were investigated (**Table 1**). Clinical diagnosis and monitoring were carried out at the National Reference Canter in Ceará state, Northeast Brazil. Leprosy diagnosis was confirmed by bacteriological analysis of slit skin smears and by histopathological examination of biopsies taken from active skin lesions.[6] At the first visit, patients had a complete dermato-neurological examination by a dermatologist with expertise in leprosy diagnosis, when the number and the body distribution of skin lesions and affected nerves were registered. Biopsy of skin lesion, venous blood and skin smear material from six sites for bacilloscopy were collected. During the clinical monitoring, patients attended the established schedule for clinical/laboratory monitoring (monthly appointment during the first year and thereafter, yearly). All patients were advised to return to an urgent appointment at the reference center in case any discomfort or new clinical manifestation appeared. In this study, the following case definitions for leprosy reactions were employed: T1R was defined as an acute clinical manifestation, usually characterized by the exacerbation of pre-existing lesions, or the appearance of new lesions. T2R was characterized by the sudden appearance of tender erythematous skin nodules (erythema nodosum leprosum/ENL) mainly accompanied by fever and other systemic symptoms such as joint pain, bone tenderness, neuritis, edema, malaise, anorexia with or without lymphadenopathy. In the clinical diagnosis of reactions, skin signs were obligatory, nerve and systemic signs were non-compulsory while neuritis, malaise, and fever could be present in both types of reaction. Treatment for leprosy reactions followed the guidelines from the Brazilian Ministry of Health. Patients with clinical manifestations not fulfilling these previously described criteria were considered suspected cases of relapses and were clinically examined by the assistant dermatologist, by the PI (GOP) and by an expert member of the independent steering committee (Dr. Sinesio Talhari). Additionally, in these patients skin smears and biopsies were collected from new lesions and used to investigate drug susceptibility (inoculation in BALB/c mice, sequencing of the *rpoB*, *folP1*, *gyrA* and *gyrB* genes and whole genome sequencing).

**Table 1:** Main demographic and clinical features of recurrent cases from the U-MDT/CT-BR trial.

Patient’s identification	1126	3208	2188
Age at diagnosis (years)	32	17	20
Sex	Male	Male	Male
Date of U-MDT (month/year)			
Start	06/2007	10/2007	09/2007
Completion	11/2007	03/2008	02/2008
Relapse date (month/year)	09/2011	04/2015	11/2014
Months between the end of U-MDT and recurrence of symptoms	46	86	79
Ridley-Jopling classification			
First diagnosis	BL	LL	LL
Relapse	LL	LL	LL
Bacillary index			
First diagnosis	4.0	4.75	3.5
Relapse	4.0	4.0	4.2
Lowest BI (months follow-up)			
	1.25	1.0	3.0
	31	43	76
Number of reactions and neuritis during follow up	4	7	3
Clinical evolution			
	Neuritis (n = 1)	Neuritis (n = 1)	
	T2R (n = 2)	T2R (n = 5)	T2R (n = 2)
	T1R (n = 1)	T1R (n = 1)	T1R (n = 1)

CRF: case report form; MDT: multidrug therapy; U-MDT: uniform MDT; T1R: BI: bacilloscopic index; type 1 reaction; T2R: type 2 reaction.

As part of the U-MDT/CT-BR trial, a well-prepared biobank of biopsies from leprosy skin lesions and serum samples, collected at various time points during treatment and monitoring, was assembled and has been properly maintained at recruitment sites and an extra back-up has been kept at the coordination center. For this study, we used skin biopsies from the first episode that were formalin-fixed and paraffin-embedded to allow long-term storage and serum samples collected at diagnosis and at various time-points during and after treatment (**Table 1 and S1 Table**). Serum IgM antibodies to *M*. *leprae-*specific PGL-1 antigen (0^.^01μg/mL NT-P-BSA) and serum IgG antibodies to the synthetic LID-1 (1μg/mL LID-1) antigen were detected by enzyme-linked immunosorbent assay (ELISA).[13, 14] Patients showing recurrent symptoms after treatment had biopsies taken from new lesions (**Table 1 and S1 Table**), which were used as the source of *M*. *leprae* for drug susceptibility testing in BALB/c mice [15] (treated with dapsone, rifampicin or no drug) and for partial [16] and whole genome sequencing.

### DNA extraction from tissue

A truXTRAC^TM^ FFPE DNA kit (Covaris) was used following the manufacturer's recommendation with some optimization. Briefly, ten 20μm FFPE tissue sections for each sample were pooled in a screw-cap microTUBE in duplicate or triplicate. Paraffin was removed and the tissue rehydrated with 100μl of tissue SDS buffer using a focused-ultrasonicator series S2 with the following settings: intensity = 5, cycles per burst = 200, time = 300s, temperature = 20°C. Digestion was done using a 40μl mixture of proteinase K (20 mg/ml) and lysozyme (10 mg/ml) using a focused-ultrasonicator with the same settings as above except for the time set at 10s. Digestion occurred at 56°C overnight followed by 1 h at 80°C to reverse the formaldehyde crosslinks. Finally, DNA was isolated from lysates using the columns of the truXTRAC FFPE DNA kit and eluted in 50μl of Covaris BE buffer. DNA was quantified using a Qubit fluorometer (ThermoFisher). For samples 1126–2011 and 2188–2014, which had been passaged in mice, DNA was extracted from mouse footpad suspensions then sheared to ~600 bp by ultrasonication and purified with AMPure beads, before library preparation.

The quantity of DNA was assessed after each critical step i.e. DNA extraction, library preparation and amplification post-array capture (**S2 Table**). Since the quality of DNA is known to be low after FFPE extraction, we did not fragment the DNA with the Covaris method as it was already fragmented nor did we size select our libraries to avoid losing too much DNA.

### Library preparation and sequencing

DNA from each extract was used to prepare Illumina libraries using a Kapa Hyper Prep kit (Kapa Biosystem) as described elsewhere.[17] To remove host DNA from the libraries, we used a custom-synthesized oligonucleotide array (Agilent) spanning the entire *M*. *leprae* genome.[18] Quality of the captured and re-amplified library was assessed using the Fragment Analyzer system (Advances Analytical technologies, Inc). The size of the captured library was 180bp and the concentration 52ng/μl.

Sequencing was performed on an Illumina Hi-Seq 2500 instrument.

### Sequence analyses

Raw reads from the same sample were merged and processed as described elsewhere [17] by adapter- and quality-trimming and alignment with the *M*. *leprae* TN reference genome (NCBI a.n. AL450380.1). To avoid false positive SNP calls the following cutoffs were applied: minimum overall coverage of 5 non-duplicated reads, minimum of 3 non-duplicated reads supporting the SNP, mapping quality score greater than 8, base quality score greater than 15 and a SNP frequency above 80%.

SNPs and short insertions and deletions (InDels) were compared between index and second episodes for each recurrent case. Unique sets of SNPs for each genome were established by comparison with the list of SNPs from 20 *M*. *leprae* genomes published elsewhere (**S3 Table**). [9, 18, 19] All unique and/or discriminatory variants were manually visualized using the IGV browser [20] to check for possible alignment inconsistencies. We additionally genotyped all samples using the SNP model described in Monot *et al*. and inferred *in silico* the VNTR copy number for 33 out of 44 known VNTR loci (11 loci were too large to be spanned with Illumina reads). [9, 11, 21]

## Results

### Demographics and diagnosis

The U-MDT/CT-BR study initially enrolled 858 patients of whom 78.4% were classified as MB. During follow-up, four of the treated patients presented with new symptoms between four and eight years after completion of U-MDT and three of these were re-investigated in this study. These participants were three young male leprosy patients (# 1126, 2188 and 3208) from Fortaleza, Ceará, Northeast Brazil, an endemic city for leprosy. The main clinical and laboratory characteristics of these three patients with recurrent signs of leprosy after U-MDT are shown in **Table 1**. In all three cases, leprosy was first diagnosed in 2007 but the patients displayed new clinical signs, which were not associated with leprosy reactions, between 2011 and 2015.

In these three patients, original leprosy skin lesions detected at diagnosis, disappeared after specific treatment and upon suspicion of relapse/reinfection, new skin lesions were observed in previously unaffected body areas. The timelines of clinical events presented by these patients during follow up (**S1 Fig**) highlight their high propensity to develop leprosy reactions, especially T2R, although all of them also developed T1R. These records also demonstrate that leprosy reactions and relapse/reinfection occurred at different time points. The timelines also illustrate the evolution of bacilloscopic index (BI) during follow up. In one case, the BI at the second episode was higher than the BI at the first episode.

In addition, the first diagnosis revealed that the three MB patients showed high IgM and IgG antibody levels to PGL-1 and LID-1 antigens, respectively (**S1 Fig**). Since these biomarkers have been used to monitor the disease state, we measured antibody levels by ELISA before, during and after U-MDT. Overall, the antibody titers gradually declined but remained above the threshold for positivity for at least one of the antigens during the study period except for patient 1126. This patient showed an antibody titer below the threshold just before the recurrence of the disease (39 months after U-MDT) and then both antibody titers increased by the time of recurrent disease. By contrast, despite oscillating levels of PGL-1 antibody for 3208, antibody titers, especially to LID-1, remained high for 3208 and 2188 during the entire study period.

### Drug susceptibility results

*M*. *leprae* from the recurrent lesions (1126–2011, 2188–2014) was inoculated into mice and only multiplied in the untreated animals, indicating that the bacilli were viable but susceptible to dapsone and rifampicin. It was not possible to inoculate mice with the sample from 3208–2015. Analysis of the *rpoB*, *folP1*, *gyrA* and *gyrB* genes revealed a wild-type sequence in all six strains, confirming susceptibility to rifampicin, dapsone, and fluoroquinolones, respectively, in all cases.

### Whole-genome analysis

Sufficient whole genome read coverage was obtained from the six *M*. *leprae* samples for genotyping and comparative genomic analyses (**S4 Table**).

**Fig 1:**
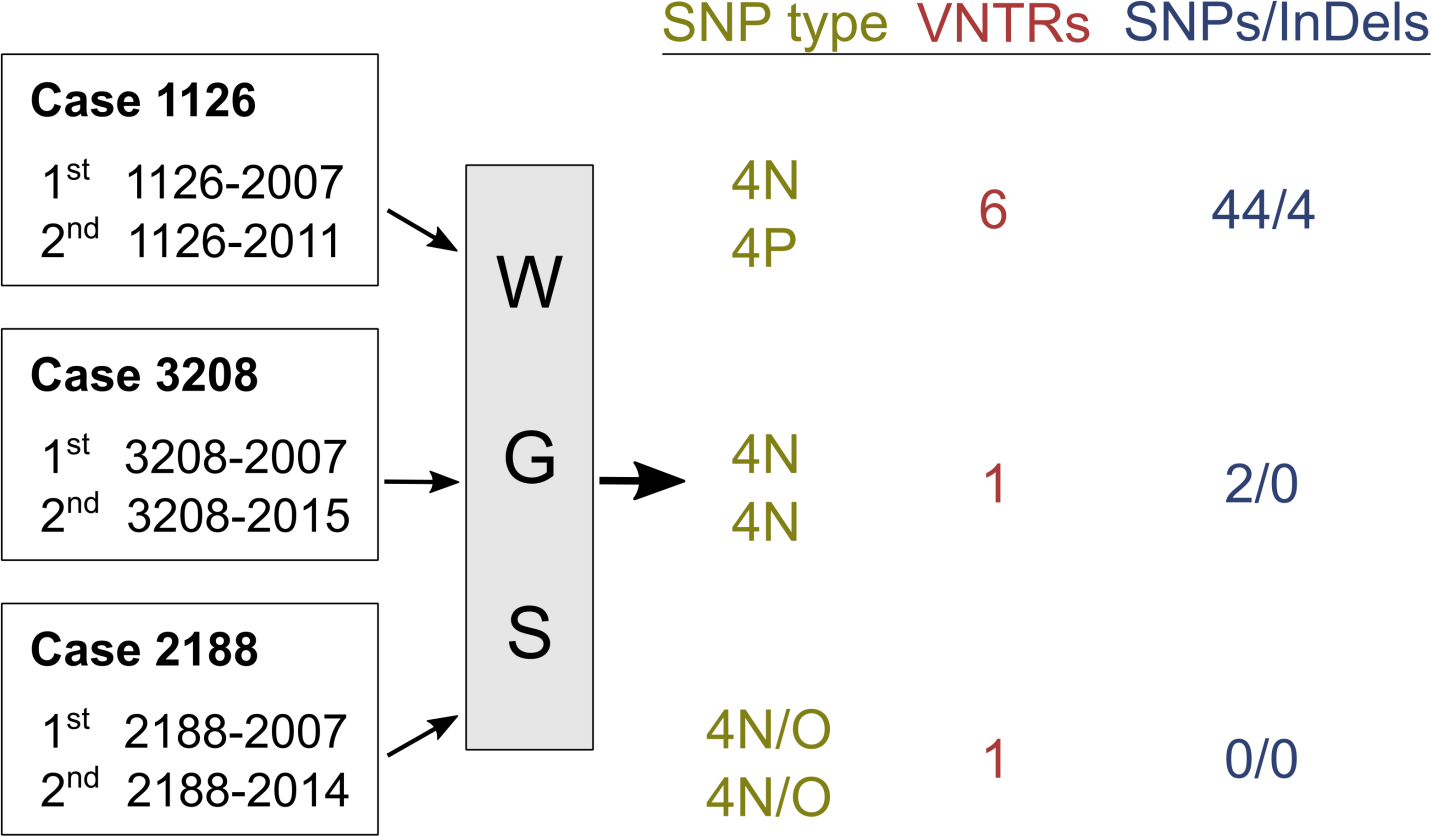
Summary of the whole genome sequencing analysis (WGS) between the *M*. *leprae* strains investigated at the 1^st^ and 2^nd^ disease occurrences.

The recurrent strain 1126–2011 was clearly distinct from the primary strain 1126–2007, and differed in 44 SNPs, 4 InDels and 6 VNTR loci (**Fig 1 and S5 Table**). Furthermore, 1126–2007 and 1126–2011 share no SNPs that might indicate close relatedness or direct ancestry.

Strains 3208–2007 and 3208–2015 differed in only two SNPs and one VNTR locus (**Fig 1 and S5 Table**). Both SNPs (T1740863C in an intergenic region and C1803024T in a pseudogene) were present in 3208–2015, indicating that 3208–2015 was certainly the direct progeny of 3208–2007. In addition, eight unique variant nucleotides were restricted to these two samples (compared to the SNPs from 20 previously published *M*. *leprae* genomes [9, 18, 19] and those from this study), confirming the identity of the strains (**S6 Table**). Interestingly, a cluster of three SNPs leads to missense mutations, in codons 495 and 496 of *asn1*, encoding an L-asparagine permease, which contributes to virulence in *Mycobacterium tuberculosis* [22].

Analysis of 2188–2007 and 2188–2014 revealed identical genome sequences (**Fig 1**). Curiously, both strains belong to a new SNP subtype intermediate between subtypes 4N and 4O. The only difference between the two genomes was found in the (GTA)9 VNTR locus (**S5 Table**), which harbored 11 repeats in 2188–2007 and 12 repeats in 2188–2014. Genome comparisons revealed that both strains share 28 unique variant nucleotides (**S7 Table**). Among them are two missense mutations in *ML0411*, encoding a PPE protein and in *ribD* (*ML1340*), the riboflavin biosynthesis protein. An insertion of 9 nucleotides (GGACATCTA at position 1,219,061) was found in *ML1052*, a putative PucR-like transcriptional regulator, which leads to a modification of the protein. Interestingly this mutation was present at only 30% frequency in 2188–2007, while it was fixed in 2188–2014.

Another frame-shift arising from a dinucleotide insertion was found in *ML0825c*, the ortholog of *rv2358* in *M*. *tuberculosis* that codes for the protein SmtB, a zinc-sensing transcriptional regulator and member of the AsrR/SmtB family.[23, 24]. The C-terminal part of SmtB is essential for the protein dimerization, zinc binding and DNA recognition. Furthermore, a specific histidine residue (H138 in ML0825c and H117 in *Synechococcus* StmB) is important for the allosteric coupling of the zinc and DNA binding sites in the protein.[25] Modeling of *M*. *leprae* StmB *in silico* (**S2 Fig**) showed that the frame-shift leads to loss of H138 and should thus impair protein function.

## Discussion

The relapse rate is considered to be the most important indicator of the efficacy of a given MDT. On the other hand, reinfection is an indicator of active transmission and the susceptibility of leprosy convalescents to new infections. This investigation provided a unique opportunity to apply high-resolution whole-genome tools to differentiate relapse from reinfection and to evaluate the impact of U-MDT on antibody levels to two *M*. *leprae* antigens. MDT affects both cellular and humoral *M*. *leprae* specific immunity. In MB patients, there is a decline in antibody levels during MDT and patients remain unable to mount a protective Th1 type immunity to *M*. *leprae* after treatment.[26] Levels of antibodies to PGL-1 and LID-1 were high in all three MB cases at diagnosis, then declined during and after treatment but nonetheless remained above the cut-off point for positivity, especially antibodies to LID-1. Our data is in accordance with previous studies showing decay in antibody titers while sero-reversion is rare in leprosy patients after regular MDT [26, 27]. By the time of recurrent disease, the antibody titers to at least one of the antigens had risen. In our study, patients were carefully monitored for treatment compliance and all completed the U-MDT treatment.

In our study the three MB patients had several episodes of leprosy reactions during follow up including T1R and mainly T2R, in accordance with the reports showing increased propensity of MB patients to develop reactions. [28, 29] In fact, several studies have shown that in some endemic areas the occurrence of T1R in BL/LL patients is higher than T2R. A study about risk factors for leprosy reactions in patients from three endemic countries (Philippines, Nepal, Brazil) showed that among all LL and BL patients, T1R was more frequent than T2R. [30] Another study from Thailand showed that T2R was slightly more prevalent than T1R in lepromatous patients. [31] T1R primarily affects immunologically unstable borderline patients (BL, BT, BB), while although sporadic, it also occurs in LL patients. T1R is characterized by an increased inflammatory Th1-type cell-mediated immunity in pre-existing skin lesions and systemically, in serum and in circulating leukocytes. The capacity of BL/LL patients, who have a predominant Th2 response, to develop T1R was elucidated by studies showing leukocytes with a Th0 profile that produce IFNγ, IL2 and IL4 or a polarized shift to Th1 type response with IFNγ and IL-12p40 mRNA in lesional skin and in leukocytes. [32, 33]

In both leprosy and tuberculosis, host genetic factors and immunological mechanisms determine the outcome of infection so that susceptibility varies among individuals. Case 1126 was unambiguously identified as reinfection because of the extensive polymorphisms between the two strains. Reinfection has long been suspected as a cause of new leprosy episodes and it has been suggested that individuals who have already had leprosy are more likely to be reinfected after treatment due to their inherent immunogenetic susceptibility.[34–36] Around 30% of relapse cases in Recife, northeast Brazil, were reported to be in contact with other leprosy patients and more often from the same family or household.[8] Leprosy case 1126 is an example of “family disease”, because both of the patient’s parents had leprosy around five years before his diagnosis, his daughter and partner had PB leprosy and the partner’s cousin, who lives in the same household, was diagnosed with MB leprosy but failed to complete MDT due to alcohol addiction.

The extremely limited genomic variability detected between strains from the same geographical origin poses a challenge in distinguishing between relapse or reinfection with a closely related strain. In a recent paper, Avanzi *et al*. showed that a strain infecting three patients in the same region of Guinea Conakry differed in only two SNPs [17] and four VNTRs. In our study, two SNPs and one polymorphic VNTR were found. While individual VNTRs carry virtually no ancestral information due to the risk of homoplasy and mutation reversion, the fact that only two SNPs were found strongly indicates that the recurrent strain was directly derived from the original infection. It should be recalled that in our study skin biopsies were taken from two different lesions in different body areas. Likewise, in the case of 2188 only one polymorphic VNTR locus distinguished between the first and the recurrent infection, and the absence of SNPs confirms the strain’s identity. Furthermore, there was no history of leprosy in either patient 3208’s or 2188’s households, and both patients had high antibody titers during the study period suggesting continued immunological stimulation by bacterial antigens after treatment. Therefore, based on the genomic analysis, the patients’ epidemiologic history and serological data, we consider that the recurrence of leprosy in both patients 3208 and 2188 was due to relapse.

Leprosy presents a variable incubation period which can range from 2–15 years. Although more prevalent in adults, leprosy also occurs in children <15 years, with reports of cases in patients younger than 1 year of age [37] indicating at least in children, short incubation period of the disease. However, nothing is known about the incubation period of reinfection, especially in genetically susceptible individuals who remain exposed to the bacilli in endemic areas. In this study, the reinfection case was observed 4 years after the conclusion of treatment, indicating a relatively short incubation period but which is in accordance with the reported range of the incubation period of the disease. The availability and larger use of whole genome sequencing studies of *M*. *leprae* in recurrent leprosy and leprosy reinfection can clarify the duration of incubation period in such cases. Further investigations of other such cases will give us a more definitive picture of characteristics of reinfection.

It is theoretically possible that the original infection in leprosy could involve more than one strain of *M*. *leprae* and, that the recurrence could be a relapse due the regrowth of one of the sub-populations of *M*. *leprae*, that had been under-treated by the first course of MDT. However, although possible, in our study this probability was implausible, since in all three patients investigated, including the reinfection case, genomic sequences of the *M*. *leprae* strains responsible for the original infections showed no mutation associated with drug resistance. Therefore, even if the original infection had involved more than one strain of *M*. *leprae*, these strains were MDT susceptible.

To conclude, this study is the first to demonstrate that it is possible to differentiate reinfection from relapse in leprosy in a field setting with a follow up period extended to eight years. This provides a proof-of-concept and emphasizes the value of whole genome sequencing in clinical follow up of leprosy. Importantly, the extended observation period allowed identification of relapses/reinfection. *M*. *leprae* grows very slowly and has a relatively long incubation time, so shorter periods of monitoring would be unlikely to provide sufficient clinical evidence to suspect relapse or reinfection. Also the two relapse cases in this study exemplify the superiority of whole-genome sequencing over genotyping a limited subset of loci or VNTR typing. For instance, the current SNP genotyping scheme can only detect distinct *M*. *leprae* lineages [9], which is not useful for analyzing closely related strains. VNTRs can distinguish such strains but do not reflect the overall genetic distance (**Fig 1**) nor convey information about strain ancestry. Improvements in sample preparation have made whole-genome sequencing more applicable routinely and we expect that recent technological advances will culminate in sequencing platforms that can be used to deliver whole genome coverage at the point of diagnosis within days of seeing the patient.[38]

All raw sequence read files have been deposited in the trace archive of the National Center for Biotechnology Information Sequence Read Archive under accession no. SRP078228.

## Supporting information

S1 Table Patient samples used for sequencing whole genome of *M*. *leprae* strains. FFPE: formalin fixed paraffin embedded skin biopsy—MFP: mouse footpad bacilli suspension.
(DOCX)

S2 Table DNA quantification after DNA extraction from the FFPE biopsy samples and MFP samples and after library preparation. FFPE: formalin-fixed paraffin-embedded; MFP: mouse foot pad; LOD: limit of detection.
(DOCX)

S3 Table List of 20 *M*. *leprae* genomes used to infer unique SNPs in recurrent cases. SNP: single nucleotide polymorphism.
(DOCX)

S4 Table Statistics of *M*. *leprae* whole genome sequences. ^1^ Fraction of total reads that aligned to the reference genome TN.
(DOCX)

S5 Table Allelic diversity of VNTR loci in the recurrent leprosy cases. VNTR: variable number tandem repeats; NA: not available because of low coverage at that locus; mixture of multiple alleles; loci where VNTR number varied between the first and second strain are highlighted.
(DOCX)

S6 Table Specific SNPs restricted to 3208–2007 and 3208–2015 strains compared to 24 other *M*. *leprae* genomes. SNP: single nucleotide polymorphism.
(DOCX)

S7 Table List of 28 unique SNPs in strains 2188–2007 and 2188–2014. SNP: single nucleotide polymorphism. ^1^At 30% frequency in 2188–2007.
(DOCX)

S1 Fig *M*. *leprae* specific anti-phenolic glycolipid 1 (PGL-1) immunoglobulin M (IgM) and anti-LID-1 IgG serology and timelines of clinical events and BI evolution of multibacillary (MB) leprosy patients that had recurrent disease after treatment. Serological data and timelines of clinical events and bacilloscopic index (BI) evolution are depicted in panels A-F: patient # 1126 (A, B); patient # 3208 (C, D); patient # 2188 (E, F). The first serum sample was collected at diagnosis before treatment and sequential samples were collected monthly during treatment and at different times during the follow-up as indicated.
(DOCX)

S2 Fig Structure and polymorphisms in SmtB. Panel A shows the sequence alignment of SmtB homologs. The locations of the α5 metal-binding sites are highlighted in blue and pink. In red is the mutated sequence of SmtB. Panel B shows the structure of the CzrA dimer from *Sthaphylococcus aureus*. Zn, in orange, binds at the interface between the two monomers. Panel C shows a model of the effect of the mutation in ML0825 on the dimer, which compromises the binding of Zn ions. The mutated part is represented in red lines. The protein was modeled using the homology modeling webserver SWISS-MODEL and the structure of the transcriptional repressor CzrA from *Sthaphylococcus aureus* (PDB code 1R1V) as template.
(DOCX)

S1 Reference list (DOCX)

## References

[1] Britton WJ, Lockwood DN. Leprosy. Lancet. 2004;363(9416):1209–19. doi: 10.1016/S0140-6736(04)15952-7 15081655 10.1016/S0140-6736(04)15952-7

[2] Han XY, Seo YH, Sizer KC, Schoberle T, May GS, Spencer JS, et al A new Mycobacterium species causing diffuse lepromatous leprosy. Am J Clin Pathol. 2008;130(6):856–64. doi: 10.1309/AJCPP72FJZZRRVMM 19019760 10.1309/AJCPP72FJZZRRVMM

[3] WHO. Global leprosy update, 2014: need for early case detection. 2015. 26343055

[4] WHO. Model Prescribing Information: Drugs Used in Leprosy. Geneva1998.

[5] Penna ML, Buhrer-Sekula S, Pontes MA, Cruz R, Goncalves Hde S, Penna GO. Primary results of clinical trial for uniform multidrug therapy for leprosy patients in Brazil (U-MDT/CT-BR): reactions frequency in multibacillary patients. Lepr Rev. 2012;83(3):308–19. . 23356032

[6] Penna ML, Buhrer-Sekula S, Pontes MA, Cruz R, Goncalves Hde S, Penna GO. Results from the clinical trial of uniform multidrug therapy for leprosy patients in Brazil (U-MDT/CT-BR): decrease in bacteriological index. Lepr Rev. 2014;85(4):262–6. Epub 2015/02/14. . 25675650

[7] Oskam L, Dockrell HM, Brennan PJ, Gillis T, Vissa V, Richardus JH. Molecular methods for distinguishing between relapse and reinfection in leprosy. Trop Med Int Health. 2008;13(10):1325–6. Epub 2008/10/22. doi: 10.1111/j.1365-3156.2008.02134_1.x TMI2134_1 [pii]. . 18937747 10.1111/j.1365-3156.2008.02134_1.x

[8] da Silva Rocha A, Cunha Dos Santos AA, Pignataro P, Nery JA, de Miranda AB, Soares DF, et al Genotyping of Mycobacterium leprae from Brazilian leprosy patients suggests the occurrence of reinfection or of bacterial population shift during disease relapse. J Med Microbiol. 2011;60(Pt 10):1441–6. Epub 2011/05/21. doi: 10.1099/jmm.0.029389-0 jmm.0.029389–0 [pii]. ; PubMed Central PMCID: PMC3347867. 21596907 10.1099/jmm.0.029389-0 PMC3347867

[9] Monot M, Honore N, Garnier T, Zidane N, Sherafi D, Paniz-Mondolfi A, et al Comparative genomic and phylogeographic analysis of Mycobacterium leprae. Nat Genet. 2009;41(12):1282–9. doi: 10.1038/ng.477 19881526 10.1038/ng.477

[10] Zhang L, Budiawan T, Matsuoka M. Diversity of potential short tandem repeats in Mycobacterium leprae and application for molecular typing. J Clin Microbiol. 2005;43(10):5221–9. Epub 2005/10/07. 43/10/5221 [pii] doi: 10.1128/JCM.43.10.5221-5229.2005 ; PubMed Central PMCID: PMC1248435. 16207987 10.1128/JCM.43.10.5221-5229.2005 PMC1248435

[11] Kimura M, Sakamuri RM, Groathouse NA, Rivoire BL, Gingrich D, Krueger-Koplin S, et al Rapid variable-number tandem-repeat genotyping for Mycobacterium leprae clinical specimens. J Clin Microbiol. 2009;47(6):1757–66. Epub 2009/04/24. doi: 10.1128/JCM.02019-08 JCM.02019-08 [pii]. ; PubMed Central PMCID: PMC2691099. 19386839 10.1128/JCM.02019-08 PMC2691099

[12] Truman RW, Singh P, Sharma R, Busso P, Rougemont J, Paniz-Mondolfi A, et al Probable zoonotic leprosy in the southern United States. N Engl J Med. 2011;364(17):1626–33. doi: 10.1056/NEJMoa1010536 21524213 10.1056/NEJMoa1010536 PMC3138484

[13] Brett SJ, Payne SN, Gigg J, Burgess P, Gigg R. Use of synthetic glycoconjugates containing the Mycobacterium leprae specific and immunodominant epitope of phenolic glycolipid I in the serology of leprosy. Clin Exp Immunol. 1986;64(3):476–83. . 2431812 PMC1542453

[14] Duthie MS, Goto W, Ireton GC, Reece ST, Cardoso LP, Martelli CM, et al Use of protein antigens for early serological diagnosis of leprosy. Clin Vaccine Immunol. 2007;14(11):1400–8. doi: 10.1128/CVI.00299-07 17898185 10.1128/CVI.00299-07 PMC2168166

[15] Shetty VP, Wakade AV, Ghate S, Pai VV, Ganapati R, Antia NH. Viability and drug susceptibility testing of M. leprae using mouse footpad in 37 relapse cases of leprosy. Int J Lepr Other Mycobact Dis. 2003;71(3):210–7. Epub 2003/11/12. doi: 10.1489/1544-581X(2003)71<210:VADSTO>2.0.CO;2 . 14608816 10.1489/1544-581X(2003)71<210:VADSTO>2.0.CO;2

[16] Kai M, Nguyen Phuc NH, Nguyen HA, Pham TH, Nguyen KH, Miyamoto Y, et al Analysis of drug-resistant strains of Mycobacterium leprae in an endemic area of Vietnam. Clin Infect Dis. 2011;52(5):e127–32. Epub 2011/02/05. doi: 10.1093/cid/ciq217 ciq217 [pii]. . 21292655 10.1093/cid/ciq217

[17] Avanzi C, Busso P, Benjak A, Loiseau C, Fomba A, Doumbia G, et al Transmission of Drug-Resistant Leprosy in Guinea-Conakry Detected Using Molecular Epidemiological Approaches. Clin Infect Dis. 2016;63(11):1482–4. Epub 2016/08/26. ciw572 [pii] doi: 10.1093/cid/ciw572 . 27558568 10.1093/cid/ciw572

[18] Schuenemann VJ, Singh P, Mendum TA, Krause-Kyora B, Jager G, Bos KI, et al Genome-wide comparison of medieval and modern Mycobacterium leprae. Science. 2013;341(6142):179–83. doi: 10.1126/science.1238286 . 23765279 10.1126/science.1238286

[19] Singh P, Benjak A, Schuenemann VJ, Herbig A, Avanzi C, Busso P, et al Insight into the evolution and origin of leprosy bacilli from the genome sequence of Mycobacterium lepromatosis. Proc Natl Acad Sci U S A. 2015;112(14):4459–64. Epub 2015/04/02. doi: 10.1073/pnas.1421504112 1421504112 [pii]. ; PubMed Central PMCID: PMC4394283. 25831531 10.1073/pnas.1421504112 PMC4394283

[20] Robinson JT, Thorvaldsdottir H, Winckler W, Guttman M, Lander ES, Getz G, et al Integrative genomics viewer. Nat Biotechnol. 2011;29(1):24–6. Epub 2011/01/12. doi: 10.1038/nbt.1754 nbt.1754 [pii]. ; PubMed Central PMCID: PMC3346182. 21221095 10.1038/nbt.1754 PMC3346182

[21] Fontes AN, Sakamuri RM, Baptista IM, Ura S, Moraes MO, Martinez AN, et al Genetic diversity of mycobacterium leprae isolates from Brazilian leprosy patients. Lepr Rev. 2009;80(3):302–15. Epub 2009/12/08. . 19961103

[22] Gouzy A, Larrouy-Maumus G, Wu TD, Peixoto A, Levillain F, Lugo-Villarino G, et al Mycobacterium tuberculosis nitrogen assimilation and host colonization require aspartate. Nat Chem Biol. 2013;9(11):674–6. Epub 2013/10/01. doi: 10.1038/nchembio.1355 nchembio.1355 [pii]. ; PubMed Central PMCID: PMC3856356. 24077180 10.1038/nchembio.1355 PMC3856356

[23] Cole ST, Eiglmeier K, Parkhill J, James KD, Thomson NR, Wheeler PR, et al Massive gene decay in the leprosy bacillus. Nature. 2001;409(6823):1007–11. doi: 10.1038/35059006 11234002 10.1038/35059006

[24] Canneva F, Branzoni M, Riccardi G, Provvedi R, Milano A. Rv2358 and FurB: two transcriptional regulators from Mycobacterium tuberculosis which respond to zinc. J Bacteriol. 2005;187(16):5837–40. Epub 2005/08/04. 187/16/5837 [pii] doi: 10.1128/JB.187.16.5837-5840.2005 ; PubMed Central PMCID: PMC1196093. 16077132 10.1128/JB.187.16.5837-5840.2005 PMC1196093

[25] Eicken C, Pennella MA, Chen X, Koshlap KM, VanZile ML, Sacchettini JC, et al A metal-ligand-mediated intersubunit allosteric switch in related SmtB/ArsR zinc sensor proteins. J Mol Biol. 2003;333(4):683–95. Epub 2003/10/22. S0022283603011306 [pii]. . 14568530 10.1016/j.jmb.2003.09.007

[26] Freitas AA, Oliveira RM, Hungria EM, Cardoso LP, Sousa AL, Costa MB, et al Alterations to antigen-specific immune responses before and after multidrug therapy of leprosy. Diagn Microbiol Infect Dis. 2015;83(2):154–61. Epub 2015/08/04. doi: 10.1016/j.diagmicrobio.2015.06.021 S0732-8893(15)00225-4 [pii]. . 26233487 10.1016/j.diagmicrobio.2015.06.021

[27] Duthie MS, Hay MN, Rada EM, Convit J, Ito L, Oyafuso LK, et al Specific IgG antibody responses may be used to monitor leprosy treatment efficacy and as recurrence prognostic markers. Eur J Clin Microbiol Infect Dis. 2011;30(10):1257–65. doi: 10.1007/s10096-011-1221-2 21544695 10.1007/s10096-011-1221-2

[28] Antunes DE, Araujo S, Ferreira GP, Cunha AC, Costa AV, Goncalves MA, et al Identification of clinical, epidemiological and laboratory risk factors for leprosy reactions during and after multidrug therapy. Mem Inst Oswaldo Cruz. 2013;108(7):901–8. doi: 10.1590/0074-0276130222 ; PubMed Central PMCID: PMC3970646. 24271045 10.1590/0074-0276130222 PMC3970646

[29] Kumar B, Dogra S, Kaur I. Epidemiological characteristics of leprosy reactions: 15 years experience from north India. Int J Lepr Other Mycobact Dis. 2004;72(2):125–33. doi: 10.1489/1544-581X(2004)072<0125:ECOLRY>2.0.CO;2 15301592 10.1489/1544-581X(2004)072<0125:ECOLRY>2.0.CO;2

[30] Scollard DM, Martelli CM, Stefani MM, Maroja Mde F, Villahermosa L, Pardillo F, et al Risk factors for leprosy reactions in three endemic countries. Am J Trop Med Hyg. 2015;92(1):108–14. Epub 2014/12/03. doi: 10.4269/ajtmh.13-0221 ajtmh.13-0221 [pii]. ; PubMed Central PMCID: PMC4347363. 25448239 10.4269/ajtmh.13-0221 PMC4347363

[31] Scollard DM, Smith T, Bhoopat L, Theetranont C, Rangdaeng S, Morens DM. Epidemiologic characteristics of leprosy reactions. Int J Lepr Other Mycobact Dis. 1994;62(4):559–67. Epub 1994/12/01. . 7868954

[32] Sreenivasan P, Misra RS, Wilfred D, Nath I. Lepromatous leprosy patients show T helper 1-like cytokine profile with differential expression of interleukin-10 during type 1 and 2 reactions. Immunology. 1998;95(4):529–36. . 9893041 10.1046/j.1365-2567.1998.00634.x PMC1364348

[33] Verhagen CE, Wierenga EA, Buffing AA, Chand MA, Faber WR, Das PK. Reversal reaction in borderline leprosy is associated with a polarized shift to type 1-like Mycobacterium leprae T cell reactivity in lesional skin: a follow-up study. J Immunol. 1997;159(9):4474–83. Epub 1997/10/31. . 9379047

[34] Pannikar V, Jesudasan K, Vijayakumaran P, Christian M. Relapse or late reversal reaction? Int J Lepr Other Mycobact Dis. 1989;57(2):526–8. Epub 1989/06/01. . 2664046

[35] Rafi A, Donoghue HD, Stanford JL. Application of polymerase chain reaction for the detection of Mycobacterium leprae DNA in specimens from treated leprosy patients. Int J Lepr Other Mycobact Dis. 1995;63(1):42–7. Epub 1995/03/01. . 7730718

[36] Brito M, Ximenes R, Gallo M. Retreatment of leprosy relapse. An Bras Dermatol. 2005;80(3):255–60.

[37] Oliveira MB, Diniz LM. Leprosy among children under 15 years of age: literature review. An Bras Dermatol. 2016;91(2):196–203. Epub 2016/05/19. doi: 10.1590/abd1806-4841.20163661 S0365-05962016000200196 [pii]. ; PubMed Central PMCID: PMC4861567. 27192519 10.1590/abd1806-4841.20163661 PMC4861567

[38] Greninger AL, Naccache SN, Federman S, Yu G, Mbala P, Bres V, et al Rapid metagenomic identification of viral pathogens in clinical samples by real-time nanopore sequencing analysis. Genome Med. 2015;7:99 Epub 2015/09/30. doi: 10.1186/s13073-015-0220-9 [pii]. ; PubMed Central PMCID: PMC4587849. 26416663 10.1186/s13073-015-0220-9 PMC4587849

