# Supplementary material for: Whole genome sequencing distinguishes between relapse and reinfection in recurrent leprosy cases
Source: PLoS Negl Trop Dis. 2017 Jun 15;11(6):e0005598. doi: 10.1371/journal.pntd.0005598 (PMC5498066; doi:10.1371/journal.pntd.0005598)
Supplement: S1 Table — FFPE: formalin fixed paraffin embedded skin biopsy—MFP: mouse footpad bacilli suspension. (DOCX) [file pntd.0005598.s001.docx]

S1 Table: Patient samples used for sequencing whole genome of *M. leprae* strains

| **Patient #** | **Type of sample for whole genome sequencing** | **Year of collection** | **Sample codes** |
| --- | --- | --- | --- |
| **1126** | FFPE | 2007 | 1126-2007 |
|  | MFP | 2011 | 1126-2011 |
| **3208** | FFPE | 2007 | 3208-2007 |
|  | FFPE | 2015 | 3208-2015 |
| **2188** | FFPE | 2007 | 2188-2007 |
|  | MFP | 2014 | 2188-2014 |

FFPE: formalin fixed paraffin embedded skin biopsy - MFP: mouse footpad bacilli suspension
