# Supplementary material for: Whole genome sequencing distinguishes between relapse and reinfection in recurrent leprosy cases
Source: PLoS Negl Trop Dis. 2017 Jun 15;11(6):e0005598. doi: 10.1371/journal.pntd.0005598 (PMC5498066; doi:10.1371/journal.pntd.0005598)
Supplement: S2 Table — FFPE: formalin-fixed paraffin-embedded; MFP: mouse foot pad; LOD: limit of detection. (DOCX) [file pntd.0005598.s002.docx]

**S2 Table: DNA Quantification after DNA extraction from the FFPE biopsy samples and MFP samples and after library preparation.**

|  | DNA concentration (ng/μl) | Volume used for library preparation (μl) | Library concentration (ng/μl) |
| --- | --- | --- | --- |
| 1126-2007_1 | <LOD | 50 | 81.7 |
| 1126-2007_2 | 0.058 | 50 | 83.4 |
| 1126-2007_3 | <LOD | 50 | 25.3 |
| 1126-2011 | <LOD | 50 | 40.9 |
| 2188-2007_1 | 0.411 | 50 | 92.9 |
| 2188-2007_2 | 0.318 | 50 | 65.1 |
| 2188-2007_3 | 8.64 | 50 | 78 |
| 2188-2014 | 0.216 | 50 | 54.4 |
| 3208-2007_1 | 0.842 | 50 | 129 |
| 3208-2007_2 | 0.136 | 50 | 145 |
| 3208-2015_1 | 20.8 | 50 | 59.2 |
| 3208-2015_2 | 25.4 | 42 | 82.9 |

FFPE: formalin-fixed paraffin-embedded; MFP: mouse foot pad; LOD: limit of detection.
