# Supplementary material for: Whole genome sequencing distinguishes between relapse and reinfection in recurrent leprosy cases
Source: PLoS Negl Trop Dis. 2017 Jun 15;11(6):e0005598. doi: 10.1371/journal.pntd.0005598 (PMC5498066; doi:10.1371/journal.pntd.0005598)
Supplement: S3 Table — SNP: single nucleotide polymorphism. (DOCX) [file pntd.0005598.s003.docx]

S3 Table: List of 20 *M. leprae* genomes used to infer unique SNPs in recurrent cases

| **Name** | **Genotype** | **Described in** |
| --- | --- | --- |
| TN | 1A | ^1^ |
| Thai53 | 1A | ^2^ |
| S2 | 1B | ^3^ |
| S11 | 1D | ^3^ |
| 3077 | 2F | ^3^ |
| Refshale_16 | 2F | ^3^ |
| SK8 | 2F | ^3^ |
| Jorgen_625 | 3I | ^3^ |
| SK2 | 3I | ^3^ |
| NHDP98 | 3I | ^4^ |
| NHDP55 | 3I | ^4^ |
| I30 | 3I | ^4^ |
| NHDP63 | 3I | ^2^ |
| S9 | 3K | ^3^ |
| Kyoto-2 | 3K | ^5^ |
| S10 | 3K | ^3^ |
| S15 | 3L | ^3^ |
| S13 | 4N | ^3^ |
| S14 | 4O | ^3^ |
| Br4923 | 4P | ^2^ |
| Mx1-22 (*M. lepromatosis*) | Outgroup | ^6^ |

SNP: single nucleotide polymorphism.
