# Supplementary material for: Whole genome sequencing distinguishes between relapse and reinfection in recurrent leprosy cases
Source: PLoS Negl Trop Dis. 2017 Jun 15;11(6):e0005598. doi: 10.1371/journal.pntd.0005598 (PMC5498066; doi:10.1371/journal.pntd.0005598)
Supplement: S4 Table — 1 Fraction of total reads that aligned to the reference genome TN. (DOCX) [file pntd.0005598.s004.docx]

S4 Table: Statistics of *M. leprae* whole genome sequences

|  | **Alignment rate (%)^1^** | **Average read depth coverage**^1^ |
| --- | --- | --- |
| **1126-2007** | 38.98 | 6_._27 |
| **1126-2011** | 23.47 | 11_._21 |
| **3208-2007** | 42.33 | 37_._19 |
| **3208-2015** | 66.41 | 31_._38 |
| **2188-2007** | 72_._36 | 123_._86 |
| **2188-2014** | 16_._74 | 58_._25 |

^1^ Fraction of total reads that aligned to the reference genome TN.
