# Supplementary material for: Whole genome sequencing distinguishes between relapse and reinfection in recurrent leprosy cases
Source: PLoS Negl Trop Dis. 2017 Jun 15;11(6):e0005598. doi: 10.1371/journal.pntd.0005598 (PMC5498066; doi:10.1371/journal.pntd.0005598)
Supplement: S5 Table — VNTR: variable number tandem repeats; NA: not available because of low coverage at that locus; mixture of multiple alleles; loci where VNTR number varied between the first and second strain are highlighted. (DOCX) [file pntd.0005598.s005.docx]

S5 Table: Allelic diversity of VNTR loci in the recurrent leprosy cases.

| **Locus ID** | **Coordinates** | **1126** | | **2188** | | **3208** | |
| --- | --- | --- | --- | --- | --- | --- | --- |
|  |  | 1126-2007 | 1126-2011 | 2188-2007 | 2188-2014 | 3208-2007 | 3208-2015 |
| (T)8, & (A)6 | 337466-337473, 337474-337479 | 8 & 6 | 8 & 6 | 8 & 6 | 8 & 6 | 8 & 6 | 8 & 6 |
| (T)6 & (N)7 & (T)8 | 514181-514186, 514187-514193, 514194-514200 | 6 & 7 & 8 | 6 & 7 & 8 | 6 & 7 & 8 | 6 & 7 & 8 | 6 & 7 & 8 | 6 & 7 & 8 |
| (A)9 | 1414666-1414674 | 8 | 8 | 8 | 8 | 8 | 8 |
| (G)9 | 976857-976865 | 9 | 10 | 9 | 9 | 9 | 9 |
| (C)9 | 2658192-2658200 | 9 | 11 | 9/10 | 9/10 | 8/9 | 8/9 |
| (G)10a | 347280-347289 | 10 | 9 | 9 | 9 | 13 | 13 |
| (G)10b | 442993-443002 | NA | ? | 9 | 9 | ? | 9/10 |
| (G)11 | 1309544-1309554 | NA | 11 | 9 | 9 | 10/11 | 12 |
| (G)12 | 1116443-1116454 | 9/10 | 10 | 9/10 | 9 | 11 | 11 |
| (CG)6 | 2947291-2947302 | 6 | 6 | 6 | 6 | 6 | 6 |
| (AC)8a | 1531185-1531200 | 9 | 8 | 8 | 8 | 9 | 9 |
| (AC)8b | 2211035-2211050 | 7 | 7/8 | 7/8 | 7/8 | 7 | 7 |
| (AC)9 | 1452573-1452590 | 8 | NA | 7/8 | 7/9 | 8 | 8 |
| (CA)6 | 2507097-2507108 | 6 | 6 | 6 | 6 | 6 | 6 |
| (TA)8 | 3221617-3221632 | 9 | NA | 10 | 10 | ? | 8/9 |
| (TA)9 | 2844971-2844988 | 7 | 6 | ? | ? | 7 | 7 |
| (TA)10 | 1744091-1744110 | 8/10 | 8/9 | 10/11 | 10/11 | 10 | 10 |
| (AT)10 | 2951821-2951840 | NA | NA | 5 | 5 | 6 | 6 |
| (AT)15 | 948935-948964 | NA | NA | ? | ? | ? | ? |
| (AT)17 | 2597735-2597768 | NA | NA | ? | 13 | 13 | 13 |
| (ACC)5 | 1980049-1980063 | 5 | 5 | 5 | 5 | 5 | 5 |
| (GGT)5 | 2567251-2567265 | 4 | 4 | 4 | 4 | 4 | 4 |
| (AGT)5a | 1237528-1237542 | 5 | 5 | 5 | 5 | 5 | 5 |
| (GTA)9 | 2583814-2583840 | 9 | 10 | 9/10 | 11 | 9 | 9 |
| (CACCG)3 | 2562391-2562405 | 3 | 3 | 3 | 3 | 3 | 3 |
| 6-3a | 1190341-1190358 | 3 | 3 | 3 | 3 | 3 | 3 |
| 6-3b | 2302531-2302548 | 3 | 3 | 3 | 3 | 3 | 3 |
| 6-7 | 1816857-1816892 | 6 | 6 | ? | 8 | 7 | 7 |
| 7-3 | 285076-285096 | 3 | 3 | 3 | 3 | 3 | 3 |
| 10-4 | 1139035-1139074 | 4 | NA | 4 | 4 | 4 | 4 |
| 12-5 | 1381661-1381723 | 5 | 5 | 5 | 5 | 5 | 5 |
| 15-3 | 2928131-2928175 | 3 | 3 | 3 | 3 | 3 | 3 |
| 27-5 | 687026-687160 | 5 | 5 | 5 | 5 | 5 | 5 |

VNTR: variable number tandem repeat; NA: not available because of low coverage at that locus; mixture of multiple alleles; loci where VNTR number varied between the first and second strain are highlighted.
