# Supplementary material for: Whole genome sequencing distinguishes between relapse and reinfection in recurrent leprosy cases
Source: PLoS Negl Trop Dis. 2017 Jun 15;11(6):e0005598. doi: 10.1371/journal.pntd.0005598 (PMC5498066; doi:10.1371/journal.pntd.0005598)
Supplement: S6 Table — SNP: single nucleotide polymorphism. (DOCX) [file pntd.0005598.s006.docx]

S6 Table: Specific SNPs restricted to 3208-2007 and 3208-2015 strains compared to 24 other *M. leprae* genomes

| **Position** | **Name** | **Type** | **TN base (ref)** | **Alternate allele** | **SNP effect** | **Amino-acid change** |
| --- | --- | --- | --- | --- | --- | --- |
|  |  |  |  |  |  |  |
| 489941 | *ML0394* | protein_coding | C | T | missense | Leu163Phe |
| 630056 | *aroB* | protein_coding | C | A | synonymous | Leu163Leu |
| 1552528 | *ansP1* | protein_coding | C | A | synonymous | Ser496Ser |
| 1552530 | *ansP1* | protein_coding | T | C | missense | Ser496Pro |
| 1552533 | *ansP1* | protein_coding | A | C | missense | Lys495Gln |
| 1785100 | *-* | Intergenic | G | A | - | - |
| 1844335 | *ML1529* | Pseudogene | C | T | - | - |
| 1900827 | *-* | Intergenic | G | A | - | - |

SNP: single nucleotide polymorphism.
