# Supplementary material for: Whole genome sequencing distinguishes between relapse and reinfection in recurrent leprosy cases
Source: PLoS Negl Trop Dis. 2017 Jun 15;11(6):e0005598. doi: 10.1371/journal.pntd.0005598 (PMC5498066; doi:10.1371/journal.pntd.0005598)
Supplement: S7 Table — SNP: single nucleotide polymorphism. 1At 30% frequency in 2188–2007. (DOCX) [file pntd.0005598.s007.docx]

S7 Table: List of 28 unique SNPs in strains 2188-2007 and 2188-2014.

| **Position** | **Name** | **Type** | **TN base (ref)** | **Alternate allele** | **SNP effect** | **Amino-acid change** |
| --- | --- | --- | --- | --- | --- | --- |
|  |  |  |  |  |  |  |
| 509409 | *ML0411* | protein_coding | G | A | missense | Asp219Asn |
| 978294 | *ML0825* | protein_coding | G | GAT | frameshift | Val128fs |
| 1219061 | *ML1052* | protein_coding | G | GGGACATCTA^1^ | codon insertion | Leu253-Val255 dup |
| 1599004 | *ML1340 / ribD* | protein_coding | C | T | missense | Gly62Asp |
| 14226 | *ML0302* | Intergenic | C | CAT | - |  |
| 46129 | *ML0038* | Pseudogene | C | T | - |  |
| 47405 | *ML0039* | Pseudogene | C | T | - |  |
| 223657 | *ML0159* | protein_coding | C | T | synonymous | Ser336Ser |
| 417955 | *PPE* | Pseudogene | C | T | - |  |
| 428724 | *-* | Intergenic | C | T | - |  |
| 534530 | *ScoA* | Pseudogene | C | T | - |  |
| 657961 | *-* | Intergenic | C | T | - |  |
| 676219 | *-* | Intergenic | C | G | - |  |
| 809458 | *-* | Intergenic | G | A | - |  |
| 1091716 | *Ag84* | protein_coding | C | T | synonymous | Leu123Leu |
| 1232532 | *ML1068* | Pseudogene | C | T | - |  |
| 1320784 | *hom* | protein_coding | C | T | synonymous | Ser381Ser |
| 1437154 | *ML1214* | protein_coding | G | A | synonymous | Thr165Thr |
| 1450750 | *-* | Intergenic | G | A | - |  |
| 1599380 | *ML1341* | Pseudogene | C | T | - |  |
| 1827911 | *-* | Intergenic | C | CG | - |  |
| 2536347 | *fprB* | protein_coding | G | A | synonymous | Arg378Arg |
| 2622952 | *mce1* | Pseudogene | AC | A | - |  |
| 2681117 | *ML2256* | Pseudogene | T | TC | - |  |
| 2878485 | *ML2407* | protein_coding | G | A | synonymous | Pro67Pro |
| 2951819 | *-* | Intergenic | GTATATATATA | G | - |  |
| 2972847 | *dnaK* | protein_coding | G | A | synonymous | Ser194Ser |
| 3187492 | *ML2661* | protein_coding | C | T | synonymous | Ser5Ser |

SNP: single nucleotide polymorphism. ^1^At 30% frequency in 2188-2007.

.
