# Supplementary material for: Whole genome sequencing distinguishes between relapse and reinfection in recurrent leprosy cases
Source: PLoS Negl Trop Dis. 2017 Jun 15;11(6):e0005598. doi: 10.1371/journal.pntd.0005598 (PMC5498066; doi:10.1371/journal.pntd.0005598)
Supplement: S1 Fig — Serological data and timelines of clinical events and bacilloscopic index (BI) evolution are depicted in panels A-F: patient # 1126 (A, B); patient # 3208 (C, D); patient # 2188 (E, F). The first serum sample was collected at diagnosis before treatment and sequential samples were collected monthly during treatment and at different times during the follow-up as indicated. (DOCX) [file pntd.0005598.s008.docx]

**S1 Fig**

**
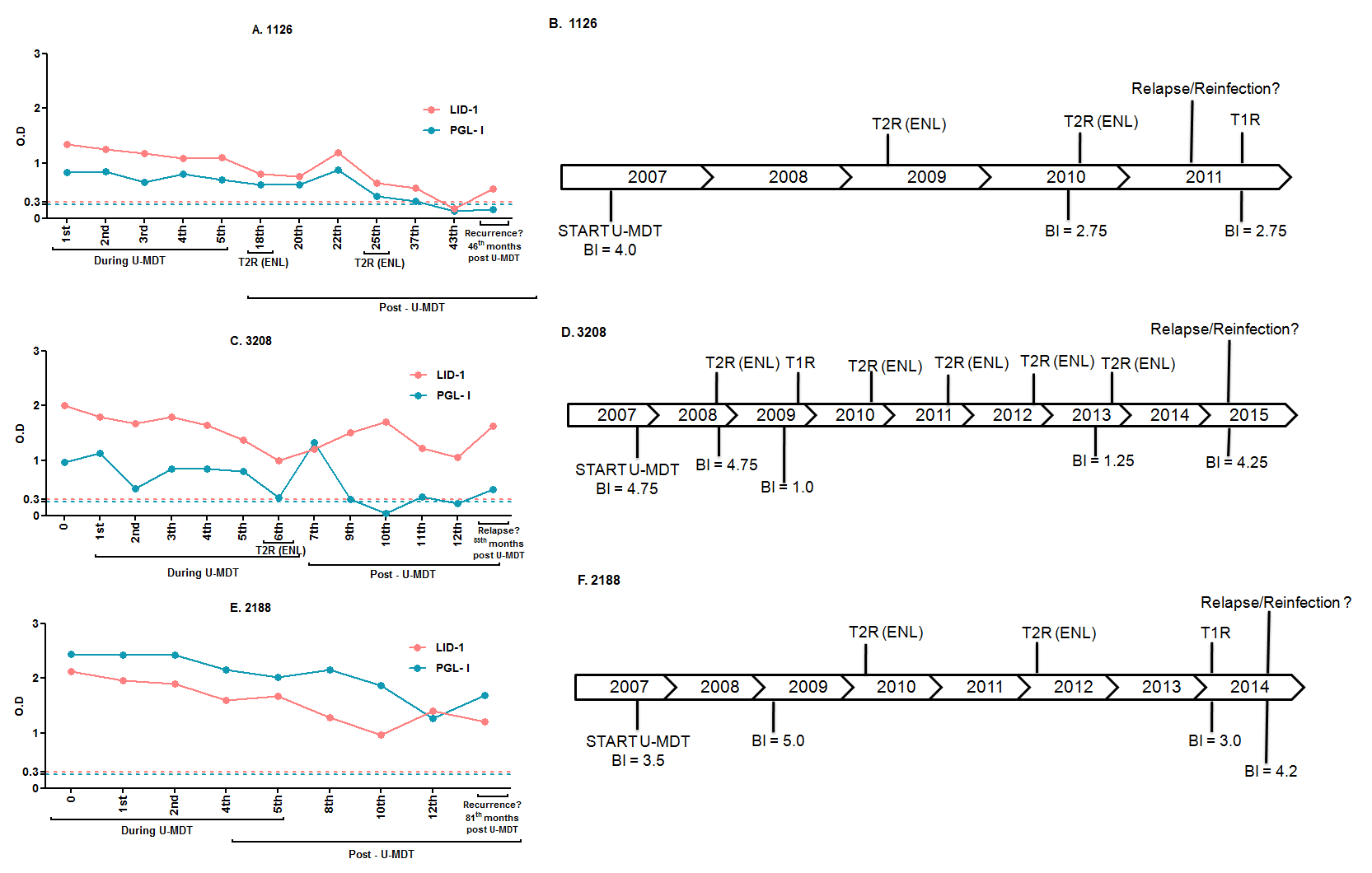
**

**S1 Fig:** *M. leprae* specific anti-phenolic glycolipid 1 (PGL-1) immunoglobulin M (IgM) and anti-LID-1 IgG serology and timelines of clinical events and BI evolution of multibacillary (MB) leprosy patients that had recurrent disease after treatment. Serological data and timelines of clinical events and bacilloscopic index (BI) evolution are depicted in panels A-F: patient # 1126 (A, B); patient # 3208 (C, D); patient # 2188 (E, F). The first serum sample was collected at diagnosis before treatment and sequential samples were collected monthly during treatment and at different times during the follow-up as indicated.
