# Supplementary material for: Whole genome sequencing distinguishes between relapse and reinfection in recurrent leprosy cases
Source: PLoS Negl Trop Dis. 2017 Jun 15;11(6):e0005598. doi: 10.1371/journal.pntd.0005598 (PMC5498066; doi:10.1371/journal.pntd.0005598)
Supplement: S2 Fig — Panel A shows the sequence alignment of SmtB homologs. The locations of the α5 metal-binding sites are highlighted in blue and pink. In red is the mutated sequence of SmtB. Panel B shows the structure of the CzrA dimer from Sthaphylococcus aureus. Zn, in orange, binds at the interface between the two monomers. Panel C shows a model of the effect of the mutation in ML0825 on the dimer, which compromises the binding of Zn ions. The mutated part is represented in red lines. The protein was modeled using the homology modeling webserver SWISS-MODEL and the structure of the transcriptional repressor CzrA from Sthaphylococcus aureus (PDB code 1R1V) as template. (DOCX) [file pntd.0005598.s009.docx]

**S2 Fig**


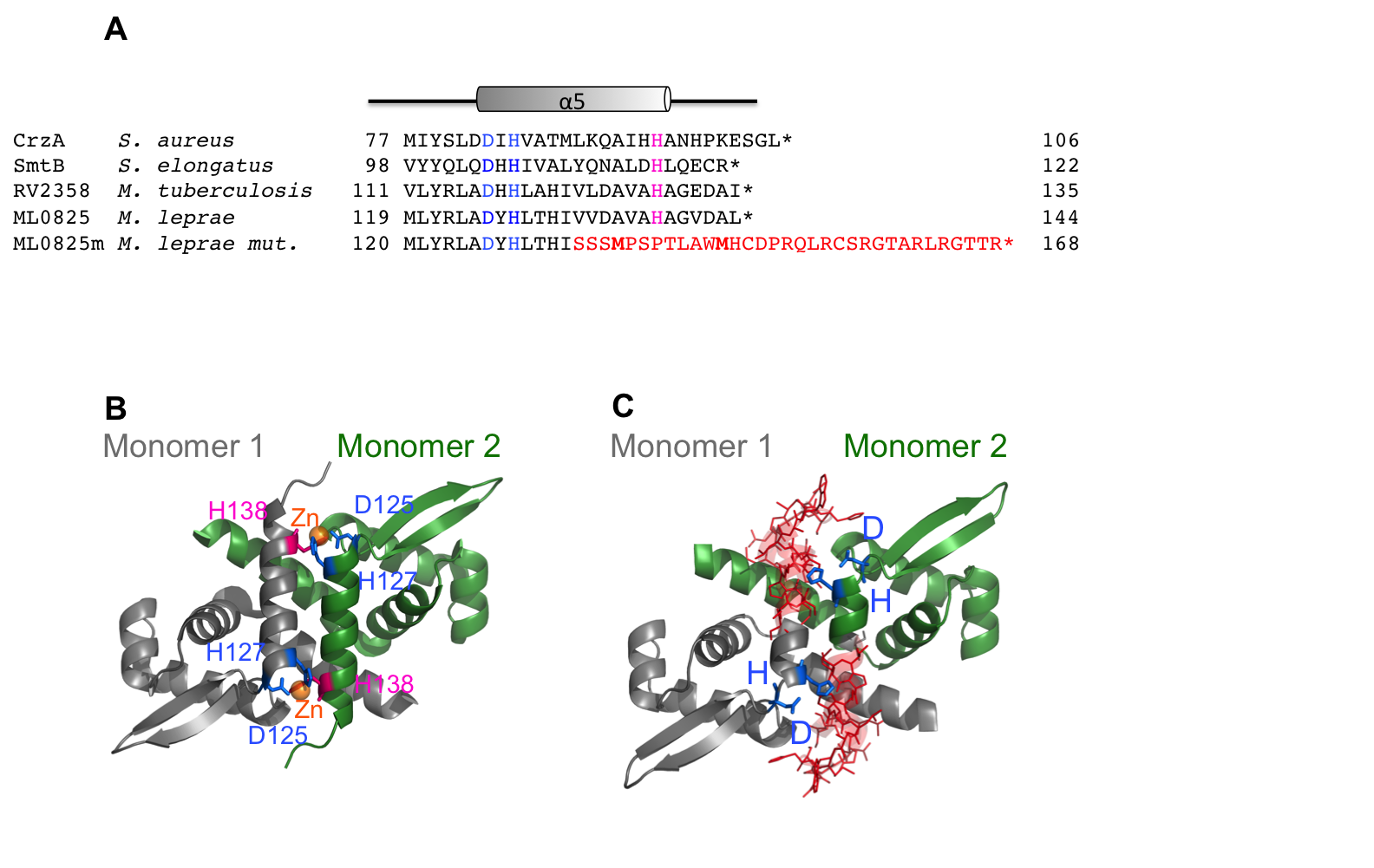


S2 Fig. Structure and polymorphisms in SmtB. Panel A shows the sequence alignment of SmtBhomologs. The locations of the α5 metal-binding sites are highlighted in blue and pink. In red is the mutated sequence of SmtB. Panel B shows the structure of the CzrA dimer from *Sthaphylococcus aureus*. Zn, in orange, binds at the interface between the two monomers. Panel C shows a model of the effect of the mutation in ML0825 on the dimer, which compromises the binding of Zn ions. The mutated part is represented in red lines. The protein was modeled using the homology modeling webserver SWISS-MODEL^7^ and the structure of the transcriptional repressor CzrA from *Staphyloccocus aureus* (PDB code 1R1V) as template.

1. Cole ST, Eiglmeier K, Parkhill J, et al. Massive gene decay in the leprosy bacillus. Nature 2001;409(6823):1007–11.

2. Monot M, Honoré N, Garnier T, et al. On the origin of leprosy. Science 2005;308(5724):1040–2.

3. Schuenemann VJ, Singh P, Mendum TA, et al. Genome-wide comparison of medieval and modern Mycobacterium leprae. Science 2013;341(6142):179–83.

4. Truman RW, Singh P, Sharma R, et al. Probable Zoonotic Leprosy in the Southern United States. N Engl J Med 2011;364(17):1626–33.

5. Kai M, Nakata N, Matsuoka M, Sekizuka T, Kuroda M, Makino M. Characteristic mutations found in the ML0411 gene of Mycobacterium leprae isolated in Northeast Asian countries. Infect Genet Evol J Mol Epidemiol Evol Genet Infect Dis 2013;19:200–4.

6. Singh P, Benjak A, Schuenemann VJ, et al. Insight into the evolution and origin of leprosy bacilli from the genome sequence of Mycobacterium lepromatosis. Proc Natl Acad Sci 2015;112(14):4459–64.

7. Bordoli L, Kiefer F, Arnold K, Benkert P, Battey J, Schwede T. Protein structure homology modeling using SWISS-MODEL workspace. Nat Protoc 2008;4(1):1–13.
