# Supplementary material for: Whole genome sequencing distinguishes between relapse and reinfection in recurrent leprosy cases
Source: PLoS Negl Trop Dis. 2017 Jun 15;11(6):e0005598. doi: 10.1371/journal.pntd.0005598 (PMC5498066; doi:10.1371/journal.pntd.0005598)
Supplement: S1 Reference list — (DOCX) [file pntd.0005598.s010.docx]

1. Cole ST, Eiglmeier K, Parkhill J, et al. Massive gene decay in the leprosy bacillus. Nature 2001;409(6823):1007–11.

2. Monot M, Honoré N, Garnier T, et al. On the origin of leprosy. Science 2005;308(5724):1040–2.

3. Schuenemann VJ, Singh P, Mendum TA, et al. Genome-wide comparison of medieval and modern Mycobacterium leprae. Science 2013;341(6142):179–83.

4. Truman RW, Singh P, Sharma R, et al. Probable Zoonotic Leprosy in the Southern United States. N Engl J Med 2011;364(17):1626–33.

5. Kai M, Nakata N, Matsuoka M, Sekizuka T, Kuroda M, Makino M. Characteristic mutations found in the ML0411 gene of Mycobacterium leprae isolated in Northeast Asian countries. Infect Genet Evol J Mol Epidemiol Evol Genet Infect Dis 2013;19:200–4.

6. Singh P, Benjak A, Schuenemann VJ, et al. Insight into the evolution and origin of leprosy bacilli from the genome sequence of Mycobacterium lepromatosis. Proc Natl Acad Sci 2015;112(14):4459–64.

7. Bordoli L, Kiefer F, Arnold K, Benkert P, Battey J, Schwede T. Protein structure homology modeling using SWISS-MODEL workspace. Nat Protoc 2008;4(1):1–13.
